# ConvLSTM-based tropical cyclone intensity estimation and classification using satellite imagery over the North Indian ocean

**DOI:** 10.1371/journal.pone.0330705

**Published:** 2025-12-05

**Authors:** Manju M. S., Harsh Pateriya, Rajeev Kumar Gupta, Deepak Singh Tomar, Punit Gupta, Asmir Butkovic

**Affiliations:** 1 Department of Computer Science and Engineering, Maulana Azad National Institute of Technology, Bhopal, Madhya Pradesh, India; 2 IIT Kharagpur, India; 3 Department of Computer Science & Engineering, Pandit Deendayal Energy University, Gandhinagar, India; 4 National College of Ireland, Dublin, Ireland; 5 Technological University Dublin, Ireland; Universiti Kebangsaan Malaysia, MALAYSIA

## Abstract

Tropical cyclones pose significant threats to coastal regions, and have a major negative influence on the environment and society. Precise cyclone identification and intensity estimation are crucial for effective early warning systems and disaster prevention. Traditional methods rely on manual interpretation and empirical models, often lacking efficiency and accuracy. This study proposes a deep learning framework that utilizes satellite image sequences for cyclone detection, classification, and intensity estimation. Unlike conventional models relying solely on spatial or manual features, the proposed hybrid architecture integrates Convolutional Neural Networks (CNNs) and ConvLSTM to learn spatiotemporal patterns jointly. Key innovations include the clustering-based cyclone region isolation method, sequence-level data augmentation, and the use of SMOTE to mitigate class imbalance. The proposed approach demonstrates substantial improvement in accuracy over baseline models, achieving 99.16% accuracy for binary classification using VGG16. An accuracy of 81.1 ± 4.33% across cyclone intensity levels, and an RMSE of 7.79 ± 1.27 knots in wind speed prediction using the ConvLSTM-based model. All models are evaluated using 5-fold cross-validation on CIMSS Tropical Data Archive and IMD Best-Track datasets. Overall, these results show an exciting potential for future use of deep learning for real time forecasting and early warning systems. Future work will also look to improve or increase model generalization, either through using ensemble learning, or potentially more complex architectures and larger datasets.

## Introduction

Among the most damaging weather events, tropical cyclones frequently cause disaster in inland and coastal regions by producing strong winds, torrential rainfall, storm surges, and widespread flooding. Since climate change has increased the frequency and strength of tropical cyclones in recent years [1,2], early warning systems, disaster planning, and mitigation activities depend heavily on the precise detection and estimation of cyclone intensity. Forecasting the intensity of cyclones is inherently complex and difficult because it demands a deep understanding of the dynamic interactions between oceanic and atmospheric systems. All these include sea surface temperature, humidity, vertical wind shear, and atmospheric pressure gradients in developing, moving, and intensifying a cyclone.

The most conventional and used traditional intensity estimation method is the Dvorak technique, used since the 1970s as a guideline in TCs assessment [3]. The Dvorak method is based on a satellite image interpretation done by means of visible and infrared determination. Meteorologists estimate intensity based on certain cloud patterns, storm eye formations, and temperature gradients. Despite its proven record for decades, the Dvorak technique relies on human skill and judgment in interpretation, allowing significant room for differences in severity classification, especially for weakly structured or rapidly intensifying cyclones [4]. It is generally less effective for cloud-filled or irregularly shaped cyclones, where the structural characteristics necessary for classification are not easily discernible. Besides the Dvorak, since then Numerical Weather Prediction [5] has also been extensively used in the cyclone intensity estimation. These models use complex mathematical equations and atmospheric physics to simulate the behaviour of cyclones according to a series of meteorological parameters, including wind speed, pressure variation, levels of humidity, and ocean temperatures. These models are very computationally intensive and thus require a capacity for high-performance computing and an extensive dataset of real-time meteorological data [5].

**Fig 1:**
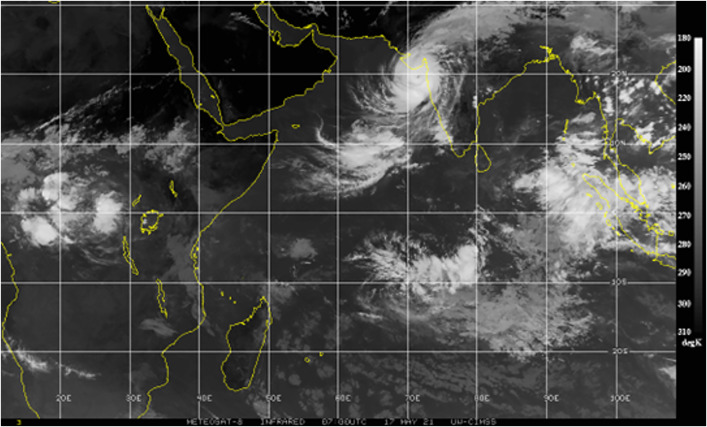
Original satellite image of a tropical cyclone sourced from the cooperative institute for meteorological satellite studies (CIMSS).

The traditional numerical models often fail to exploit the spatial and temporal patterns in the satellite imagery to the fullest extent possible, thereby limiting their predictability. Deep learning and data-driven approaches have transformed many aspects of meteorology, such as cyclone detection and intensity estimation [6]. CNNs are particularly well-suited for analysing high-resolution satellite images of tropical storms since they have demonstrated remarkable success in automated image analysis [7]. Fig 1 shows an example of a satellite image from the CIMSS tropical cyclone data archive, highlighting complex cloud structures associated with a tropical cyclone. Such features serve as critical inputs for training spatial models like CNNs. Unlike traditional methods that rely on handcrafted features and manual interpretation, CNNs automatically extract hierarchical spatial features from cyclone images, allowing them to differentiate cyclone structures from non-cyclone atmospheric formations with minimal human intervention [8]. This automation reduces subjectivity, increases consistency, and enhances efficiency. Therefore, CNN-based models are highly valuable for operational cyclone forecasting. However, several challenges persist in deep learning-based approaches. One of the major issues is that generalizability models trained on cyclone data from one region (e.g., the North Indian Ocean) may not perform well on cyclones from other basins (e.g., Atlantic or Pacific) due to regional differences in cyclone morphology and environmental conditions [9].

Additionally, interpretability remains a challenge, prompting the development of explainable AI (XAI) techniques to make these models more transparent and trustworthy to meteorologists [10]. While this study does not directly incorporate XAI techniques, its development underscores the importance of model interpretability in operational forecasting. Several recent studies have attempted to incorporate deep learning for TC intensity estimation. Many existing models either ignore temporal dependencies or treat sequential image frames independently, limiting their ability to capture evolving storm dynamics. Furthermore, existing approaches often neglect challenges such as image resolution variability, missing frames, and dataset imbalance, which are prevalent in real-world cyclone data.

This study aims to estimate and classify the intensity of tropical cyclones using deep learning models, with a focus on the north indian ocean region, a cyclone-prone area with densely populated coastlines, where timely forecasts are crucial for minimizing human and economic losses. The primary objectives include: Binary classification of cyclone and non-cyclone satellite images. Multi-class classification of cyclones into predefined intensity levels and intensity estimation to predict wind speeds. To achieve these objectives, the research work proposes a novel deep learning architecture that combines convolutional layers for spatial feature extraction with ConvLSTM layers for modelling temporal evolution. The proposed methodology addresses several challenges, including class imbalance, noisy backgrounds, and data scarcity in sequential satellite imagery.

The unique contributions and novelty of this research work are:

1. Spatiotemporal Feature Learning: Jointly capture spatial and temporal patterns, improving intensity estimation over models using either alone.
2. Cyclone Region Isolation: A clustering-based method isolates the cyclone area by reducing background noise to enhance feature relevance.
3. Sequence-Level Augmentation: Temporally-consistent data augmentation to better preserve sequence integrity during training.
4. Class Imbalance Handling: The impact of imbalanced cyclone grade classes is addressed using the SMOTE technique, improving classification robustness.
5. Hybrid Architecture: The model architecture combines CNNs for spatial analysis and ConvLSTM for temporal dynamics, offering better representational efficiency than standalone models.

The findings of this study have significant implications for early warning systems, disaster response strategies, and operational meteorology, contributing to the development of more accurate and automated cyclone classification models. The rest of this paper is structured as follows: The related works section reviews related works and existing approaches. The methodology section presents the dataset and methodology used in this work. The result and discussion section includes details experimental results and discussions. The future scope and challenges sections contain the limitations, along with directions for future research. Finally, the conclusions section concludes the study by summarizing the main findings, contributions, limitations, and potential future directions.

## Related works

There are many traditional approaches to forecasting tropical cyclones (to include the Dvorak method [3] and numerical weather prediction (NWP) [5] models) which have been very successful in many respects for operational meteorology, but they also come with several limitations associated with their core assumptions. One major issue is that many of these methods, like the Dvorak method, are predicated upon subjective interpretation/analysis, and this can and does introduce intra- and inter-analyst biases [11,12]. Moreover, these methods have challenges that make it difficult to understand qualitatively what ‘real’ tropical cyclone behavior is where the highly complex, nonlinear, and time-dependent nature of tropical cyclones is concerned. For instance, with the proof-of-concept work using mixed methods, forecasting of rapid intensification and structural evolution requires analysis of short time scales not adequately addressed by Dvorak or NWP methods, an analytical gap which is substantive by previous research [13]. Since these methods have limited generalization that spans multiple basins because of some limitations in data collection and meteorological dynamics, they simply don’t work as well outside of their training areas [14]. Additionally, traditional methods fail to provide the spatial-temporal resolution that a reliable real-time intensity estimate would require [15,16]. These limitations make a transition to machine learning (ML) and deep learning (DL) approaches for addressing intensity forecast issues especially relevant in tropical cyclone analysis, as these techniques are designed to handle high-dimensional datasets, temporal dependence, and various environmental conditions of storms.

Recent advancements in deep learning techniques, in particular CNNs and recurrent neural networks (RNNs), have facilitated the estimation of tropical cyclone (TC) intensity: C. Zhang et al. (2021) [12] proposed a TC intensity estimation model called TCICENet which seamlessly combines classification (of TC’s) and CNN-based regression on infrared (IR) satellite images; the results achieved RMSE 8.60 kt and MAE 6.67 kt, which outperformed traditional studies and previous DL models. Sattar et al. (2025) [17] made a GRU (Gated Recurrent Unit) model that used high-resolution meteorological data that spans 37 isobaric (pressure) levels, achieved MAE 4.16, 34.35% better than the mean absolute error of the Saf-Net. These models illustrate the advantages brought by spatial and temporal aspects of the data, which help to provide a better tropical cyclone intensity classification estimation. To fill in gaps of either purely data-driven or physics-based models, hybrid models have been explored.

Varalakshmi et al. (2023) [18] combined CNN models (AlexNet, VGG16) with regressors (e.g., SVR – Support Vector Regressor, and Linear Regression) and achieved RMSE values of 4.88 kt. Z. Ma et al. (2024) [19] presented a dual-attention model based on an Xception backbone, which fused multi-scale IR and water vapor images with a Laplacian pyramid fusion and attention modules and achieved 11.4% improvement in RMSE and 8% improvement in MAE. These multi-branch, multimodal models provide a balance of accuracy and complexity, which shows potential for operational forecasting. Transformer-based models and attention mechanisms were explored and improved TC intensity estimation due to their ability to capture long-range dependencies and complex spatial-temporal patterns. Zhao et al. (2025) [15] introduced TFA-Net, a dual-branch transformer net with gated feature fusion on sequential satellite images, resulting in an RMSE of 7.21 kt, and R² of 0.93. Tian et al. (2024) [20] developed ViT-TC, a vision transformer with multitask learning, outperforming CNN baselines with MAE 6.49 kt. Zhang et al. (2025) [21] proposed STIA, integrating spatial-to-temporal and temporal-to-spatial attention modules on Himawari-8 image sequences, achieving RMSE 3.61 m/s and MAE 2.83 m/s, surpassing state-of-the-art models by over 10% in RMSE reduction. These works demonstrate transformers’ potential for precise and responsive TC intensity prediction.

Multi-task learning (MTL) and cross-basin generalization remain critical challenges. Ding et al. (2024) [22] proposed CBIL-TCIE, a cross-basin incremental learning model predicting maximum sustained wind (MSW) and minimum sea-level pressure (MSLP), mitigating catastrophic forgetting and improving performance by 19.2% over fine-tuning. Zhao et al. (2024) [23] developed MT-GN, integrating shared CNNs with graph-based task embedding to learn task relationships and spatial correlations, achieving 90.37% classification accuracy and RMSE 9.50 kt. These highlight MTL and graph learning as key to robust, transferable TC forecasting. Multi-source data fusion also advances TC estimation accuracy. Xu et al. (2024) [24] proposed FHDTIE, combining satellite imagery and reanalysis data via clustering, U-Net, and graph convolutional networks, achieving lowest RMSE and MAE across 10 datasets. W. Tian et al. (2024) [25] introduced TC-Rolling, fusing multi-source satellite imagery with deviation-angle variance (DAV) via 3D CNN and Convolutional Block Attention Module, reporting RMSE 4.48–13.94 kt and outperforming baselines. W. Tian et al. (2024) [25] introduced TC-Rolling, fusing multi-source satellite imagery with deviation-angle variance (DAV) via 3D CNN and Convolutional Block Attention Module, reporting RMSE 4.48–13.94 kt and outperforming baselines.

Generative models and augmentation are used to address data scarcity. Pang et al. (2021) [26] incorporated DCGAN and YOLOv3 to synthetically augment data. The study achieved 97.78% classification accuracy and 81.39% average precision. Ibrar et al. (2025) [13] applied an LSTM autoencoder and Gaussian filters to detect disturbances in estuarine environments. The tests produced MSE 0.0359, illustrating the ability of unsupervised methods like autoencoders for anomaly detection. Regional models enhance local applicability. Mawatwal and Das (2024) [14] incorporated CNN and YOLO and trained a hybrid architecture based on images from North Indian Ocean satellite data. The resulting model achieved 98.4% accuracy for cyclone detection and 63.83% accuracy for intense classifying of five categories with RMSE 16.2 kt. Pal et al. (2024) [27] proposed Small Skip Net (SSN) to use as a lightweight model where skip connections and residual blocks mitigated vanishing gradients and reduced data load. In testing, SSN achieved 92.35% classification accuracy on 80 bytes of small imagery data from INSAT-3D satellite images, outperforming deeper networks, also offered for real-time operations. Previous ML model types also exhibited effectiveness while being more interpretable than deep learning due to features available to the study models. For example, Kar and Banerjee (2021) [28] demonstrated Random Forest classifiers were trained from a combination of geometric IR satellite features achieved 86.66% accuracy; model correctly classified more than Dvorak. Kar et al. (2019) [11], incorporated multilayer perceptrons and used statistics extracted from spatiotemporal aspects of the satellite to classify intensities with 84% accuracy.

The incorporation of physical models with ML builds upon previous approaches to improve performance. Niu et al. (2025) [29] created a hybrid model by linking the ML-based Pangu with the physics-based WRF model through spectral nudging along with a data assimilation approach that used observed FY-4B satellite data, leading to an overall improvement of 20.3% for typhoon track forecasts, and a reduction of 12.5% typographical intensity error. Cheng et al. (2025) [16] implemented a convolutional neural network (CNN) model with layers that were rotation-invariant and multi-frame satellite motion, achieving improvements of RMSE and Mean Absolute Error > 22%. Forecasting of rapid intensification (RI) was still problematic. Sharma et al. (2025) [30] used SMOTE to develop an interpretable support vector machine (SVM) model on aspects of the SHIPS dataset achieving a Probabilities of Detection of 0.88 and Heidke Skill Score of 0.492 for the Indian Ocean, equaling the SHIPS-RII-C model, which illustrated the interest in ML and the potential for RIs to be detected with ML. Regardless we propose several limitations to the body of research. Many studies fail to compare their models using standardized datasets for the community to compare the performance. Furthermore, while older methods are often criticized, the majority of works do not appropriately create a barrier of entry to compare old vs new deep learning models. The supply of models that embody lightweight and operationally feasible outcomes that are between low-to-high fidelity – like the event-based super-resolved model (SSN) proposed by Pal et al. (2024) [27] – is sparse. Further, there is little discussion regarding whether interpretability will always be feasible or not, (e.g., most transformer and hybrid CNN-ML frameworks lack interpretability).

In conclusion, the literature from 2019 to 2025 reflects a clear shift from manual and shallow-learning methods to complex, data-driven deep learning approaches. These include multi-task learning, attention mechanisms, transformer architectures, and physically informed modeling strategies. The summary of this methodology is given in Table 1 categorized by approach. Each method is evaluated based on its technique, key advantages, and limitations, providing insight into trends and research gaps in cyclone forecasting. While these methods have led to substantial gains in performance, challenges related to benchmarking, explainability, computational efficiency, and generalization across basins remain open avenues for future research. Bridging these gaps is essential for transitioning from high-accuracy research models to reliable, real-time operational forecasting systems.

**Table 1:** Summary of methodologies in cyclone intensity estimation (2019–2025).

Methodology Type	Representative Studies	Technique used	Advantage	Limitation
Traditional Methods	Dvorak Technique, NWP [3,5]	Manual interpretation, rule-based analysis	Operationally established, interpretable	Subjective, low spatial-temporal resolution, basin-specific limitations
CNN & RNN-based Deep Learning	TCICENet [12], GRU-based model [17]	CNNs, residual blocks, 3D CNNs, GRU-based sequence modeling	Learns spatiotemporal patterns; outperforms traditional models	High computational cost; limited explainability
Hybrid CNN + ML Models	Varalakshmi et al. (2023) [18], Z. Ma et al. (2024) [19]	CNN + SVR/LR, attention fusion, multi-branch networks	Enhances accuracy via multimodal fusion and classic ML interpretability	Limited generalization; fusion complexity
Transformer & Attention-based Models	TFA-Net [15], ViT-TC [20], STIA [21]	Vision Transformer, attention fusion, spatial-temporal interactions	Captures long-range dependencies, saliency-aware learning	Resource-intensive; interpretability and real-time viability concerns
Multi-Task Learning (MTL)	CBIL-TCIE [22], MT-GN [23]	Shared CNNs, cross-basin incremental learning, graph-based task modeling	Simultaneous MSW & MSLP prediction; avoids forgetting when adapting	Requires more data and training complexity
Multi-source Fusion Models	FHDTIE [24], TC-Rolling [25]	Satellite + reanalysis fusion, U-Net, CBAM, GCN	Integrates spatial/temporal/environmental features	Fusion design can be ad hoc; difficult to optimize weights

## Methodology

**Fig 2:**
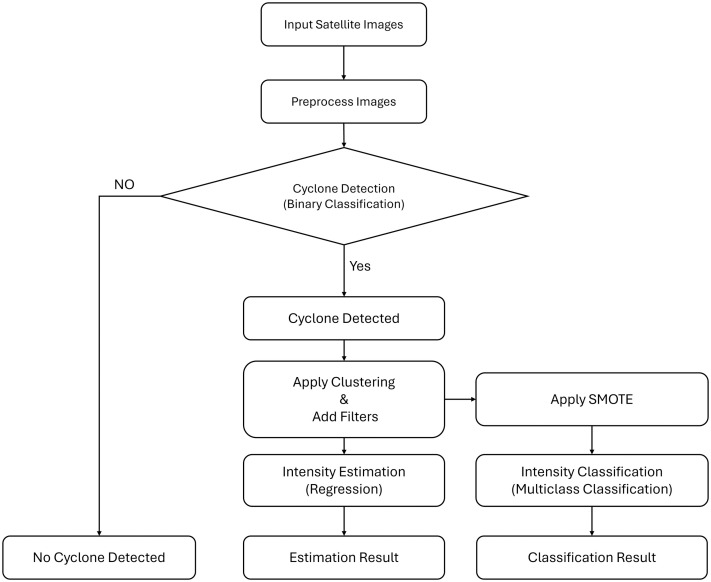
Workflow diagram illustrating the complete cyclone intensity estimation and classification framework proposed in this study.

The methodology begins by inputting and preprocessing satellite images to enhance quality and prepare data for analysis. A binary classification model is then used to detect the presence of a cyclone. If no cyclone is detected, the process ends; otherwise, detected cyclone images undergo clustering and filtering to refine the data. For detected cyclones, the system performs two parallel tasks: intensity estimation using a regression model and intensity classification using a multiclass classifier, where SMOTE is applied to handle class imbalance. The process outputs both estimation and classification results for further meteorological analysis. Fig 2 illustrates this systematic approach for cyclone intensity estimation and classification using satellite imagery.

### Data collection and source

The satellite (long wave Infrared) images are collected from the University of Wisconsin – (CIMSS, https://tropic.ssec.wisc.edu/) [31]. Satellite images of 15 tropical cyclones of the North Indian Ocean focusing on the last 10 years of data (2013–2022) are selected for the study. The dataset provides a spatial resolution of 10 km, with a temporal resolution of 3 or 6 hours for continuous monitoring of tropical cyclone development and progression. The IMD Cyclone Best Track Data provided by the India Meteorological Department (IMD) through the (https://mausam.imd.gov.in/) [32], maintained by the Regional Specialized Meteorological Centre (RSMC) in Delhi is used for labelling the intensity. The best track data contains information on tropical cyclone parameters, including latitude, longitude, central pressure, and maximum sustained wind speed, recorded at regular intervals. The cyclone intensity measurements are provided with a resolution of 5 knots to ensure accurate classification and analysis of different storm categories.

### Data preprocessing

The satellite images undergo preprocessing to enhance feature extraction and classification accuracy. The steps include:

1. Cropping & Resizing: Each image is cropped to focus on the cyclone region and resized to 310 × 310 pixels.
2. Gray Scaling: Converts images to a single-channel format, reducing computational complexity.
3. Gaussian Blur: Applied to smooth pixel variations and remove noise.
4. Erosion: Enhances cyclone boundary visibility by reducing small cloud artifacts.

**Fig 3:**
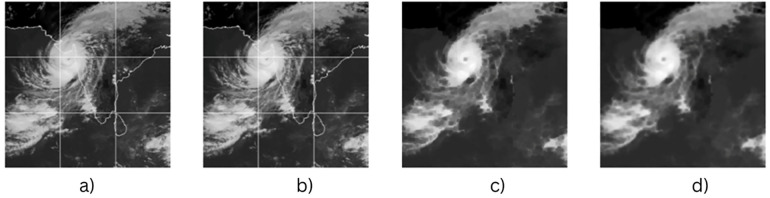
(a) Original satellite image of a cyclone, (b) resized version (310 × 310 pixels), (c) eroded image highlighting key features, and (d) Gaussian-blurred image used for noise reduction.

These preprocessing steps aim to reduce background noise and enhance model robustness. Fig 3 illustrates the workflow: (a) the cyclone region is initially cropped to focus on the indian ocean only, (b) the satellite images were manually cropped and resized to 310 × 310 pixels while preserving a 1:1 aspect ratio. This specific resolution helps exclude the cyclone eye from the centre, enhancing model robustness by incorporating more varied, non-cyclonic regions. It strikes a practical balance between spatial detail and computational efficiency, as larger sizes increase memory usage and training time without meaningful performance improvement. Therefore, 310 × 310 was selected as an effective input size for capturing spatiotemporal cyclone patterns. (c) Morphological erosion is applied to remove grid lines, artificial borders, and other high-contrast artifacts commonly present in satellite images. These elements do not carry meaningful meteorological information and can mislead the model during training. d) Gaussian blur is used to smooth the image, reducing high-frequency noise and helping the model focus on broader spatial patterns rather than isolated pixel-level variations. Together, these steps enhance the quality of input data and support more stable and generalizable learning. The dataset contains a total of 1,200 images, which are split into training and testing sets using an 80−20 ratio. The split is stratified based on cyclone intensity levels to ensure that each class is proportionally represented in both subsets. To enhance the training dataset, data augmentation techniques such as rotation, horizontal flipping, and zooming are applied, following the approach described in [33].

### Binary classification of cyclones

The binary classification (cyclone vs. non-cyclone), utilizes transfer learning with VGG16, ResNet50, and InceptionV3 [34–36]. These models are chosen because:

- VGG16: Captures fine-grained spatial details.
- ResNet50: Mitigates vanishing gradient issues through residual connections.
- InceptionV3: Utilizes multi-scale feature extraction for better pattern recognition.

**Fig 4:**
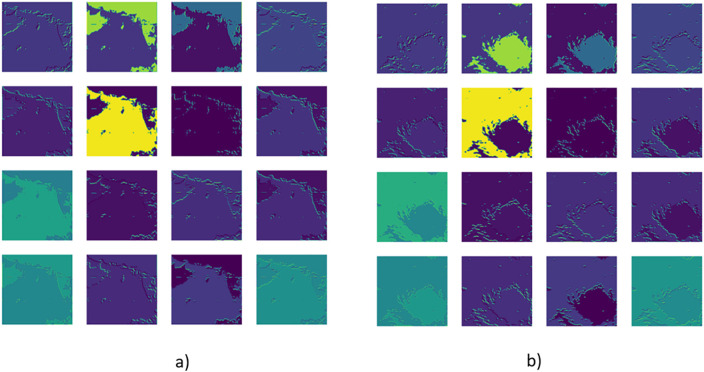
Feature maps from the first convolutional layer (Conv1) showing activations for (a) a non-cyclone image and (b) a cyclone image, illustrating early layer feature extraction differences.

Algorithm 1 describes the training procedure of the transfer learning-based cyclone classification model, making use of pretrained CNNs like VGG16, ResNet50, or InceptionV3. The model takes advantage of extracting spatial features from the pretrained models, while only tuning the last few for the cyclone task. The models retain their convolutional layers while replacing the output layer with a fully connected layer (256 neurons), dropout layer, and sigmoid output. Training is performed using Adam optimizer (LR = 0.0001) for 40 epochs with batch size 32. Performance is evaluated using accuracy, precision, recall, F1 score, and a confusion matrix [37]. Fig 4 shows the feature maps extracted from the first convolutional layer (Conv1) of the VGG16 model. (a) shows a non-cyclone image, while (b) depicts a cyclone image. These maps reveal how the model captures low-level spatial features like edges and cloud formations, essential for cyclone detection. Different filters highlight distinct patterns, such as outer cloud bands or the cyclone’s eye [34]. The varying activation intensities indicate that VGG16’s initial layers focus on edge detection and texture representation, forming the basis for deeper layers to learn cyclone-specific features.

Algorithm 1. Training of transfer learning-based cyclone classification.

**Table:** 

Initialize pretrained CNN M (VGG16, ResNet50, InceptionV3) with frozen convolutional base, retaining spatial feature extraction.	
1	**Load & preprocess** dataset $D=\;\{\left(X_{i},Y_{i}\right){\}}_{i=\text{1}}^{N}$, where X represents cyclone/non-cyclone images, and Y represents labels. Resize X to 224×224, normalize X←X/255.0, augment using random rotation, flipping, zooming, and split into $D_{train}$,$D_{val}$
2	**Extract hierarchical features** via $F_{M}(X)$, flatten convolutional outputs from layer L, and pass through dense layers with ReLU activation, dropout p=0.5, and batch normalization
3	**Attach classifier** C(x with fully connected layers, ReLU activations, and final sigmoid activation for binary classification.
4	**Define loss function** $L=-\frac{\text{1}}{N}\sum_{i=\text{1}}^{N}y_{i}\text{log}\hat{y_{i}}+\left(\text{1}-y_{i}\right)\text{log}\left(\text{1}-\hat{y_{i}}\right)$, optimize using Adam ($\alpha ={\text{1}\text{0}}^{-\text{4}},\beta_{\text{1}}=\text{0}.\text{9}$, $\beta_{\text{2}}=\text{0}.\text{9}\text{9}\text{9}$).
5	**Train model** for T epochs using mini-batch SGD (batch size m=32), fine-tuning last k convolutional layers, employing early stopping and checkpointing the best weights based on validation loss.

### Cyclone segmentation techniques

To isolate cyclone regions from satellite images, various clustering techniques are applied:

- K-Means Clustering
- DBSCAN (Density-Based Spatial Clustering of Applications with Noise) [38]
- Agglomerative Clustering
- Gaussian Mixture Models (GMM)
- Mean Shift

A comparative analysis is conducted to determine the most effective segmentation technique, using the silhouette score to measure performance based on cohesion and separation. Texture-based feature extraction methods further enhance segmentation accuracy and reduce background noise. Gabor filtering detects swirling cyclone structures by convolving the grayscale image with orientation-specific kernels [39], while Local Binary Pattern (LBP) captures fine-grained texture variations to distinguish the cyclone from surrounding cloud formations. The Canny edge detector enhances intensity gradients, refining the cyclone’s boundary [40].

**Fig 5:**
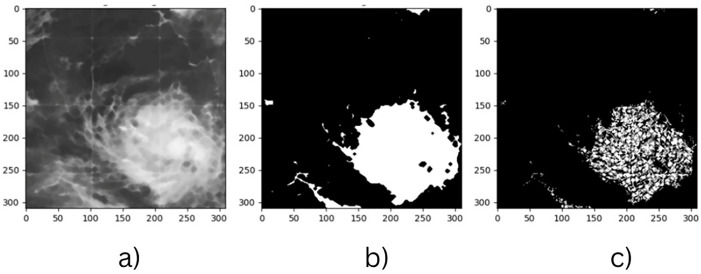
(a) Pre-processed satellite image, (b) K-Means clustered segmentation output, and (c) feature-based mask highlighting cyclone-relevant regions extracted.

To achieve precise segmentation, the extracted feature maps are combined with the K-Means mask using pixel-wise logical operations. Morphological filtering is used to remove small cloud artifacts and noise. Techniques such as opening (erosion followed by dilation) eliminate minor unwanted regions while closing (dilation followed by erosion) ensures cyclone structures remain intact, minimizing false positives and improving segmentation reliability. Fig 5 illustrates this process: (a) the original satellite image, (b)K-Means segmentation output, (c) the feature-based mask integrating texture and edge features. The feature mask, refined using Gabor filtering, LBP, and Canny edge detection, ensures better segmentation and is used for training classification models. After segmentation, contour detection identifies the cyclone’s ROI, with the largest contour representing the cyclone. Furthermore, classification models trained on labelled cyclone data categorize the cyclone as a tropical depression, storm, or severe cyclone [41].

### Cyclone intensity estimation and classification

The proposed cyclone intensity and classification framework is designed to estimate and categorize multiple cyclone intensity levels using sequences of satellite images and a deep spatiotemporal learning model. The architecture integrates TimeDistributed convolutional layers with ConvLSTM, forming a hybrid model tailored to the spatiotemporal nature of cyclone evolution. ConvLSTM networks are particularly suited for spatiotemporal data, where both the spatial structure and their temporal evolution are critical. Traditional CNNs or LSTMs, when used in isolation, fail to simultaneously capture these joint dependencies. The ConvLSTM layer, by applying convolutional operations within the recurrence, allows the model to learn motion-aware spatial features across sequential frames, an essential aspect of tropical cyclone dynamics. The process begins by providing a cyclone intensity label to categorical text in the dataset. It is numerically encoded using label encoding to make it compatible with the softmax-based classification output. To model temporal patterns of cyclone evolution, images are grouped into sequential frames, where each sample consists of four consecutive satellite images. Because we are taking satellite images with a six-hourly gap, only four satellite images will be there for a day. The target label for a sequence corresponds to the cyclone intensity of the last frame in that sequence. To enhance the model’s ability to generalize and learn robust spatial features, frame-wise data augmentation is performed [33].

Specifically, each frame in a sequence undergoes random transformations independently. These include horizontal flipping, rotations up to 30 degrees, zooming, and width/height shifts of up to 30%. This technique allows the model to learn features invariant to orientation, scale, and position, while preserving the temporal structure of the cyclone evolution across sequences. Unlike standard augmentation approaches applied only once per image, this frame-wise augmentation is embedded directly into the preprocessing pipeline, increasing the diversity of samples while maintaining sequence integrity. To ensure generalization and minimize bias, the model is evaluated using 5-fold cross-validation and is averaged across folds for both classification and estimation. Early stopping is applied to terminate training once convergence is detected, reducing the risk of overfitting.

**Fig 6:**
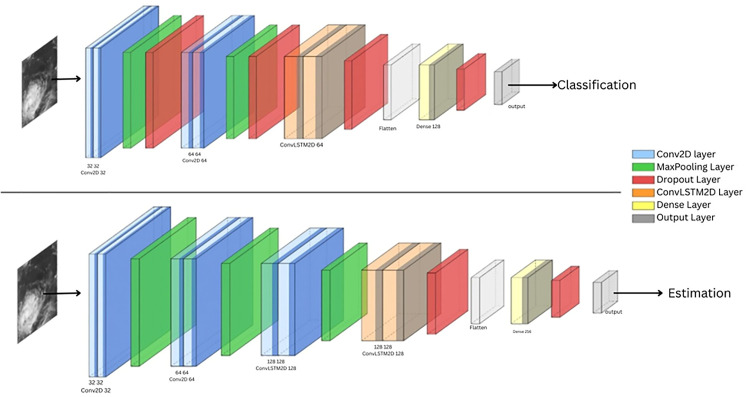
Schematic block diagram of the ConvLSTM-based deep learning architecture employed for cyclone intensity estimation and classification.

As shown in Fig 6, the estimation model begins by extracting spatial features using TimeDistributed Conv2D layers, which apply convolutional operations independently to each frame in a four-image sequence. Table 2 describes the Layer-wise comparison of the ConvLSTM-based architectures used for cyclone wind speed estimation and intensity classification. Differences in convolutional depth, filter size, and dropout rates highlight trade-offs in model complexity and target task. The estimation network includes three convolutional layers with 32, 64, and 128 filters (3 × 3 kernels, ReLU activation), each followed by 2 × 2 max pooling and dropout layers (rates of 0.2, 0.3, and 0.4, respectively) to downsample feature maps and reduce overfitting. These frame-level features are passed to a ConvLSTM2D layer with 128 filters and a 3 × 3 kernel, which captures both spatial patterns and their temporal evolution. Unlike traditional LSTMs, ConvLSTM retains spatial structure, enabling it to detect motion cues like spiral rainbands or eye development. The output from the ConvLSTM layer is flattened and passed through a Dense layer with 256 units and a 0.5 dropout rate, followed by a final output neuron that predicts the normalized wind speed. The model is trained using the Huber loss function, chosen for its robustness to outliers, and optimized with Adam (learning rate = 0.001).

**Table 2:** Model architecture comparison of estimation vs classification.

Layer Index	Layer Type	Estimation Model	Classification Model
1	TimeDistributed Conv2D	Filters: 32, Kernel: (3,3), Activation: ReLU	Filters: 32, Kernel: (3,3), Activation: ReLU, L2(0.01)
2	TimeDistributed MaxPooling2D	Pool Size: (2,2)	Pool Size: (2,2)
3	TimeDistributed Dropout	Rate: 0.2	Dropout (not TimeDistributed), Rate: 0.3
4	TimeDistributed Conv2D	Filters: 64, Kernel: (3,3), Activation: ReLU	Filters: 64, Kernel: (3,3), Activation: ReLU, L2(0.01)
5	TimeDistributed MaxPooling2D	Pool Size: (2,2)	Pool Size: (2,2)
6	TimeDistributed Dropout	Rate: 0.3	Dropout (not TimeDistributed), Rate: 0.4
7	TimeDistributed Conv2D	Filters: 128, Kernel: (3,3), Activation: ReLU	*Not included*
8	TimeDistributed MaxPooling2D	Pool Size: (2,2)	*Not included*
9	TimeDistributed Dropout	Rate: 0.4	*Not included*
10	ConvLSTM2D	Filters: 128, Kernel: (3,3), Activation: ReLU	Filters: 64, Kernel: (3,3), Activation: ReLU, L2(0.01)
11	Dropout	Rate: 0.5	*Not included*
12	Flatten	Yes	Yes
13	Dense	Units: 256, Activation: ReLU, L2 Regularization	Units: 128, Activation: ReLU, L2(0.01)
14	Dropout	Rate: 0.5	Rate: 0.5
15	Output Dense	Units: 1 (Regression)	Units: len(label_encoder.classes_), Activation: Softmax (Classification)

Algorithm II. Training of ConvLSTM for Cyclone Intensity estimation.

**Table:** 

Initialize model parameters θ,ω, learning rate α, batch size m, penalty coefficient λ, and Adam optimizer hyperparameters β1,β2.	
1	Load dataset $D=\{\left(x_{i},y_{i}\right){\}}_{i=\text{1}}^{N}$, where $x_{i}$ represents input images and $y_{i}$ represents wind speeds.
2	Normalize wind speeds y=StandardScaler(y)
3	Construct time-series sequences $S_{t}=\{x_{t-k},\ldots ,x_{t}\}$
4	**For** k=1,2,…,N **do**
5	** For** $t=\text{1},\text{2},\ldots ,n_{\text{critic}}$ **do**
6	** For** i=1,2,…,m **do**
7	Sample a sequence $S_{t}\sim p(S)$ and corresponding wind speed label $y_{t}$.
8	Pass $S_{t}$ through TimeDistributed CNN layers with ReLU activations and dropout regularization.
9	Extract temporal features using ConvLSTM with 3D kernels.
10	Flatten features and pass through fully connected dense layers.
11	Compute loss $L^{\left(i\right)}=\text{HuberLoss}\left(y_{\text{pred}},y_{t}\right)$
12	** End For**
13	Update critic parameters $\omega \leftarrow \text{Adam}\left(\nabla_{\omega }\frac{\text{1}}{m}\sum_{i=\text{1}}^{m}L^{\left(i\right)},\omega ,\alpha ,\beta_{\text{1}},\beta_{\text{2}}\right).$
14	** End For**
15	Sample new mini-batch $\{S_{t}^{\left(i\right)}{\}}_{i=\text{1}}^{m}$
16	Update generator parameters $\theta \leftarrow \text{Adam}\left(-\nabla_{\theta }\frac{\text{1}}{m}\sum_{i=\text{1}}^{m}L^{\left(i\right)},\theta ,\alpha ,\beta_{\text{1}},\beta_{\text{2}}\right)$
17	** End For**
18	Store the best model weights based on validation loss.
19	Train until early stopping criteria is met.

Algorithm II describes the training process in detail, including sequence sampling, feature extraction, and parameter updates. A known challenge in cyclone datasets is class imbalance, where certain intensity classes may be underrepresented. To mitigate this, the Synthetic Minority Over-sampling Technique (SMOTE) is applied to the training data [42]. Before resampling, image sequences are reshaped into flat feature vectors. SMOTE then generates synthetic examples for the minority classes to balance the class distribution. After resampling, the data is reshaped back into the original temporal sequence format suitable for deep learning.

**Fig 7:**
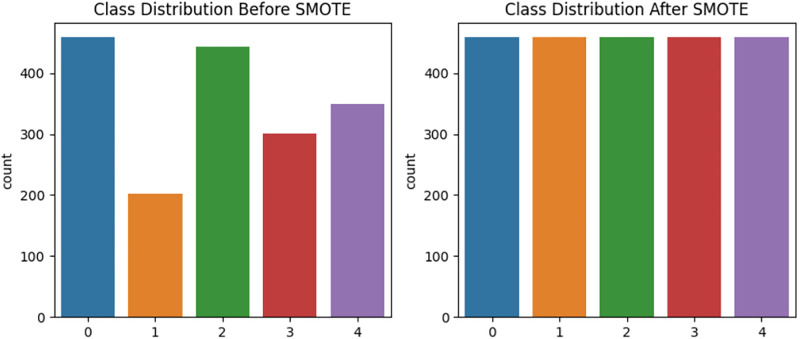
Class distribution of cyclone intensity categories before and after applying SMOTE to the training dataset.

Fig 7 shows the class distribution before and after applying SMOTE in this study, showing how class imbalance among cyclone intensity categories is corrected through synthetic oversampling. After data augmentation and before applying SMOTE, the class distributions were 451, 202, 450, 306, and 347. After applying SMOTE, each class was balanced to 451 samples. The classification model shares a similar architecture with the estimation model, with key differences in depth and complexity. It includes only two TimeDistributed Conv2D layers (with 32 and 64 filters, respectively), compared to the deeper convolutional stack in the estimation model. Additionally, the ConvLSTM2D layer in the classification network uses 64 filters, whereas the estimation model employs 128 filters. These modifications reduce the model’s complexity while preserving its ability to capture essential spatiotemporal features required for classifying cyclone intensity. The output of the ConvLSTM is flattened and passed to a dense layer with 128 neurons and ReLU activation, followed by a dropout layer with a rate of 0.5. Finally, a softmax output layer maps the output to multiple cyclone intensity classes.

Algorithm III. Training of ConvLSTM for Cyclone Intensity Classification

**Table:** 

Input: $\{X_{n},y_{n},g_{n}\},$ where n=1,2,…,N; hyperparameters α,λ,β maximum epochsE Output: Trained model f(X;θ)	
1	Initialize θ randomly
2	Load dataset $\{X_{n},y_{n}\}$ from source and apply preprocessing $P\left(X_{n}\right)$, including resizing, normalization, and augmentation
3	Encode labels $y_{n}$ using label mapping $L\left(y_{n}\right)$
4	Construct temporal sequences $S_{t}=\{X_{t}^{\left(i\right)},y_{t}^{\left(i\right)}{\}}_{i=\text{1}}^{M}$ with time stepsT
5	Split $S_{t}$ into training set $D_{train}$ and testing set $D_{test}$ using stratified sampling
6	Reshape $X_{n}$ into (M,T,H,W,C) format for ConvLSTM processing
7	**while** θ not converged & maximum epochs E not reached **do**
8	**for** each mini-batch $B\subset D_{train}$ **do**
9	Pass B through ConvLSTM model: $c_{t}=\sigma \left(W_{c}*X_{t}+b_{c}\right)$ for convolutional layers $h_{t}=\sigma \left(W_{h}*c_{t}+U_{h}*h_{t-\text{1}}+b_{h}\right)$ for LSTM layers
10	Compute loss L(θ) as sparse categorical cross-entropy with regularization: $L(\theta )=-\sum_{i}y_{i}\text{log}\left(\hat{y_{i}}\right)+\lambda {\left||\theta |\right|}^{\text{2}}$
11	Compute gradient $\nabla_{\theta L}(\theta )$ via backpropagation
12	Update model parameters using Adam optimizer: $\;\;\;\;\;\;\theta \leftarrow \theta -\alpha \nabla_{\theta L}(\theta )$
13	**end for**
14	Compute validation loss $L_{val}(\theta )$ on $D_{test}$
15	Apply learning rate scheduler and early stopping conditions if necessary
16	**end while**
17	Evaluate final model f(X;θ) on $D_{test}$ using accuracy, precision, recall, and F1-score
18	Return trained model θ

Algorithm III outlines the procedure for training the ConvLSTM model for cyclone intensity classification. It includes input preprocessing, sequence construction, data augmentation, loss computation, and performance evaluation. Training is performed using the Adam optimizer with a learning rate of 0.0005. The loss function is sparse categorical cross-entropy, which is regularized with L2 penalties to prevent overfitting. Early stopping (patience = 3) and a learning rate reduction mechanism (factor = 0.5, patience = 2) are used to optimize training convergence [43]. Training occurs over a maximum of 50 epochs with a batch size of 32. All experiments were executed on a high-performance computing (HPC) cluster using the max_dgx queue with a job configuration of 1 node, 10 CPUs, and 2 NVIDIA GPUs on a DGX. The allocated GPUs ensured efficient training and evaluation of the deep learning model, with hardware availability and utilization verified dynamically during runtime.

## Results and discussion

### Binary classification of cyclone

**Fig 8:**
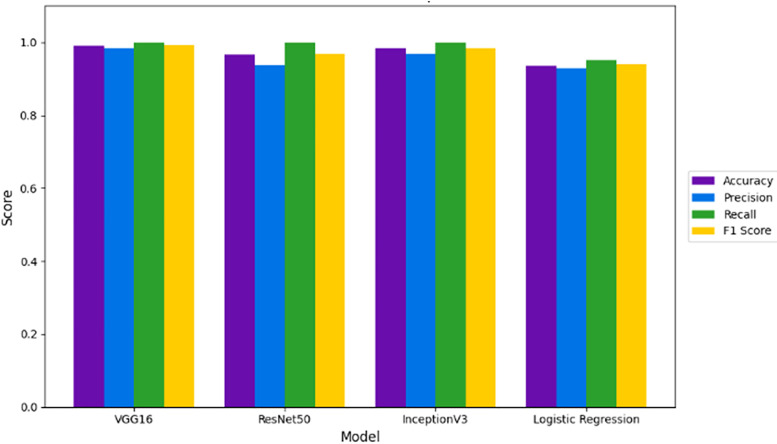
Comparative performance of different models for binary cyclone classification.

The binary classification of cyclone intensity was performed using three pre-trained deep learning models, VGG16, ResNet50, and InceptionV3, alongside a classical baseline model, Logistic Regression. Fig 8 illustrate a visual comparison across different models used for binary classification of cyclone vs. non-cyclone images. VGG16 outperforms others, highlighting its strong feature extraction capability for cyclone detection. Table 3 provides Binary classification performance of different models including VGG16, ResNet50, InceptionV3, and Logistic Regression. VGG16 achieves the highest accuracy and F1 score, demonstrating its effectiveness in distinguishing cyclone from non-cyclone imagery. VGG16 achieved the highest accuracy (99.16%), followed by InceptionV3 (98.33%) and ResNet50 (96.67%), whereas Logistic Regression obtained a lower accuracy of 93.52%. The deep learning models significantly outperformed the baseline, demonstrating their superior ability to capture complex spatial patterns in cyclone imagery. While ResNet50 introduces residual learning, its performance slightly lags due to its complex architecture, which may require more data for optimal generalization [44].

**Table 3:** Performance metrics of various models for binary cyclone intensity classification.

Model	Accuracy	Precision	Recall	F1 Score
VGG16	0.991667	0.983607	1.0	0.991736
ResNet50	0.966667	0.937500	1.0	0.967742
InceptionV3	0.983333	0.967742	1.0	0.983607
Logistic Regression	0.9352	0.93	0.95	0.94

### Clustering algorithm performance in cyclone segmentation

**Fig 9:**
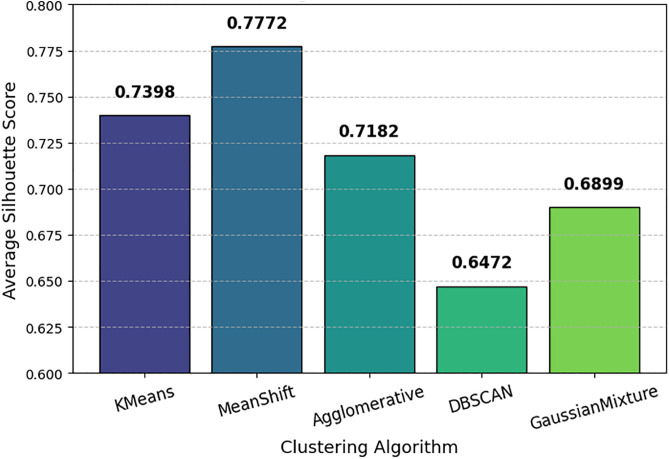
Comparison of clustering algorithms (K-Means, MeanShift, etc.) based on the average Silhouette Score.

A comparative analysis of different clustering algorithms K-Means, Mean Shift, Agglomerative Clustering, DBSCAN, and Gaussian Mixture, based on their average Silhouette Scores is done and shown in Fig 9. Average Silhouette Scores for different clustering algorithms used in cyclone region isolation. Mean Shift achieved the highest score, indicating superior cluster separation and cohesion for segmenting cyclone-relevant regions in satellite imagery. K-Means followed with a score of 0.7398, showing strong clustering performance. Agglomerative Clustering and Gaussian Mixture had moderate scores of 0.7182 and 0.6899, respectively. DBSCAN performed the worst with a score of 0.6472, suggesting that it may struggle with clear boundary definition in cyclone segmentation. This comparison helps in selecting the most effective clustering technique for cyclone feature extraction.

### Cyclone intensity estimation and classification model evaluation

Both K-Means and MeanShift clustering algorithms were employed to preprocess the dataset before estimation and classification, and their impact on performance was evaluated.

**Fig 10:**
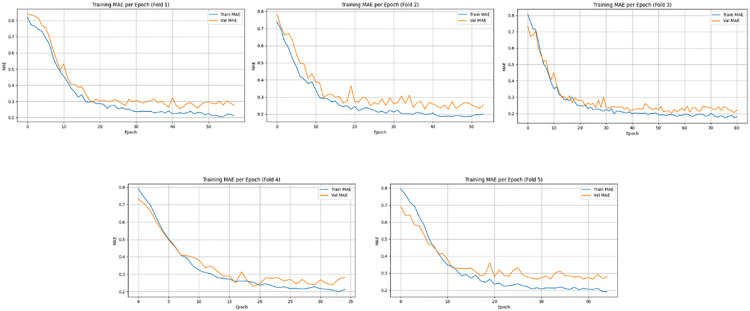
Training and validation Mean Absolute Error (MAE) per epoch across five-fold cross-validation using the MeanShift clustered dataset.

**Fig 11:**
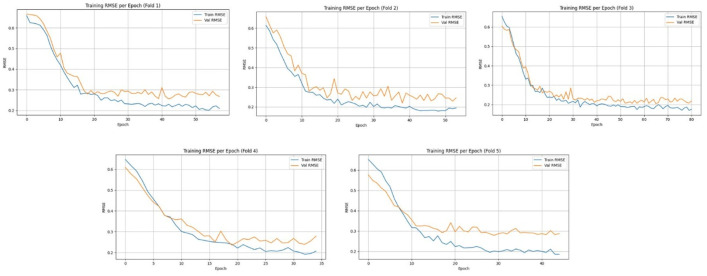
Training and validation Root Mean Square Error (RMSE) per epoch across five-fold cross-validation using the MeanShift clustered dataset.

Fig 10 and Fig 11 present the training and validation error curves - Mean Absolute Error (MAE) and Root Mean Square Error (RMSE), respectively across 5-fold cross-validation on the Meanshift dataset. In both Figs, the error consistently decreases across epochs for each fold, indicating effective convergence of the model during training. The validation curves closely follow the training trends in most folds, suggesting good generalization with minimal overfitting. While minor fluctuations in validation metrics are observed, especially in MAE plots for folds 2 and 5, the overall trend confirms stable model learning and robustness across all folds. The model achieved an average RMSE of 8.31 ± 1.10 over five folds, with a total training time of 9742.57 seconds, reflecting both the computational cost and predictive accuracy of the proposed spatiotemporal deep learning framework.

**Fig 12:**
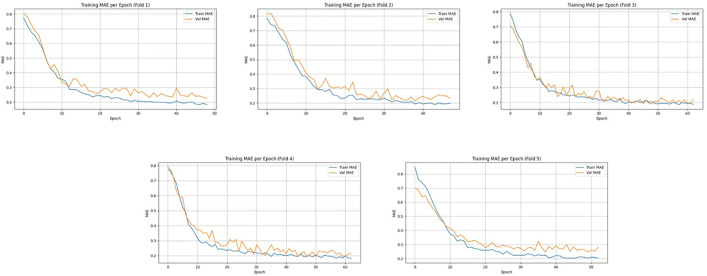
Training and validation MAE per epoch across five-fold cross-validation using the K-Means clustered dataset.

**Fig 13:**
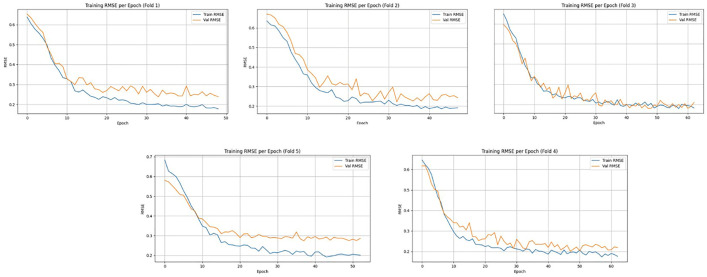
Training and validation RMSE per epoch across five-fold cross-validation using the K-Means clustered dataset.

Fig 12 and Fig 13 illustrate the training and validation MAE and RMSE per epoch, respectively, across 5-fold cross-validation for the enhanced experimental setup. In all five folds, both MAE and RMSE show a consistent decreasing trend, indicating effective model convergence and generalization. The close alignment between training and validation curves suggests that the model is not overfitting and is robust across different validation splits. Minor fluctuations in the validation RMSE, especially in folds 2 and 5, are within acceptable bounds and highlight the natural variability in temporal satellite-based intensity estimation. The model achieved an average RMSE of 7.79 ± 1.27 with a total training time of 7743.23 seconds, demonstrating improved performance and computational efficiency over prior configurations.

Based on the experimental results, the K-Means-based framework outperformed the MeanShift-based setup, achieving a lower average RMSE of 7.79 ± 1.27 compared to 8.31 ± 1.10. Additionally, K-Means required less training time (7743.23 sec) than MeanShift (9742.57 sec), highlighting its computational efficiency. While MeanShift is more adaptive to complex cluster shapes, K-Means proved more effective and stable for cyclone region isolation in this context. Hence, K-Means was selected as the optimal clustering approach for the proposed model.

**Fig 14:**
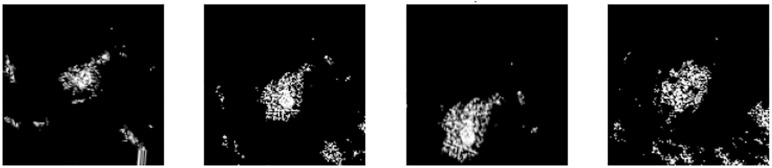
Example of misclassification, showing four consecutive cyclone images where the true class (Cyclonic Storm, CS) was incorrectly predicted as Very Severe Cyclonic Storm (VSCS).

**Fig 15:**
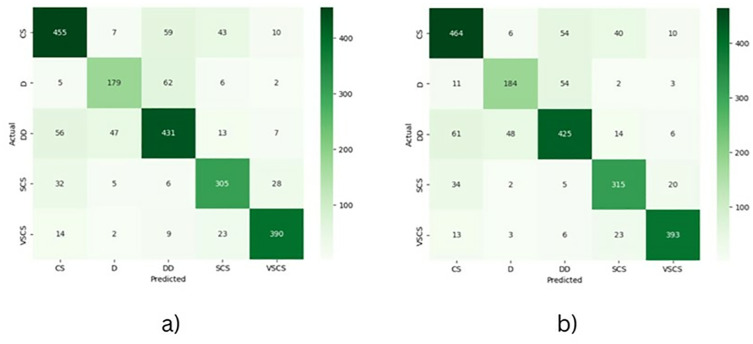
Confusion matrices illustrating cyclone intensity classification performance using (a) the K-Means clustered dataset and (b) the MeanShift clustered dataset.

The MeanShift-clustered dataset achieved slightly higher average accuracy (0.8110) than the KMeans-based approach (0.8015), suggesting it forms more effective clusters for classification tasks. However, this came at the cost of a longer average training time per fold (1776.18s vs. 1504.80s). Despite having more misclassified samples (106 vs. 94), MeanShift demonstrated better class separation, especially in the confusion matrix for D and SCS classes. On the other hand, K-Means had fewer misclassifications and trained faster, making it more computationally efficient but slightly less accurate. Fig 14 shows a misclassified sample from the K-Mean cluster dataset where the true class is ‘CS’ (Cyclonic Storm), but the model predicted ‘VSCS’ (Very Severe Cyclonic Storm). The evaluation results are summarized in Table 4, and corresponding confusion matrices are presented in Fig 15 a) (KMeans) and Fig 15 b) (MeanShift). The confusion matrix for K-Means (Fig 15 a) shows better precision for the CS and DD classes, while MeanShift (Fig 15 b) improves classification for D and SCS, suggesting it handles more ambiguous classes slightly better. Yet, MeanShift also demonstrates a higher tendency toward class confusion, particularly between neighboring intensity levels like D and DD. MeanShift is preferable when accuracy and deeper class separation are the priorities, ideal for applications requiring high reliability in cyclone prediction. K-Means is a better choice when efficiency and lower training overhead are critical, such as in time-sensitive or resource-constrained forecasting systems.

**Table 4:** K-Means vs. Meanshift clustered datasets for cyclone intensity classification.

Metric	KMeans	MeanShift
Misclassified Samples	94	106
Accuracy (Avg over 5 folds)	0.8015 ± 0.0154	0.8110 ± 0.0433
Training Time (Fold 5)	1254.05 seconds	1319.94 seconds
Avg Training Time per Fold	1504.80 seconds	1776.18 seconds

The proposed spatiotemporal deep learning framework demonstrated superior performance in both cyclone intensity estimation and classification tasks compared to baseline models. While MeanShift achieved a slightly higher classification accuracy (81.10%) than K-Means (80.15%), it incurred greater training time and more misclassifications, particularly in regions with ambiguous class boundaries. K-Means proved to be more computationally efficient and more balanced in precision across multiple classes, making it ideal for time-sensitive applications. Table 5 describes the performance comparison of the proposed framework against baseline models (LSTM, GRU, CNN) for both cyclone intensity classification and wind speed estimation. The proposed model outperforms others in classification accuracy and estimation error (RMSE), achieving the highest classification accuracy (81.1 ± 4.33) and competitive estimation RMSE (8.31 ± 1.10 RMSE). These results confirm the effectiveness of the proposed hybrid framework in capturing cyclone dynamics with higher precision and robustness, making it a strong candidate for operational tropical cyclone analysis systems.

**Table 5:** Performance comparison of the proposed framework with baseline architectures.

Sl.no	Architecture	Intensity classification	Intensity estimation
1	Proposed	81.1 ± 4.33	7.79 ± 1.27
2	LSTM	68.18 ± 4.65	9.35 ± 1.9
3	GRU	69.09 ± 4.6	8.18 ± 0.7
4	CNN [14]	63.83 ± 1.3	16.2 ± 0.9

### Future scope and challenges

In this study, one of the principal issues is the relatively small dataset (1,200 images) which restricts a model’s capability to learn robust and generalizable spatiotemporal features. This problem is significant for rare or extreme cyclone intensity classifications, as greater representation is required due to their relatively low frequency to avoid biased learning and subsequently poor classification performance. Moreover, increased risk of overfitting is another consequence of limited representation, particularly for deep learning models which rely heavily on large datasets. To partially mitigate this limitation, image augmentation techniques such as rotation, zoom, and flipping were applied to artificially expand the training dataset and introduce variability. However, further dataset expansion through data synthesis remains necessary.

While deep learning models have drastically improved cyclone intensity estimation and classification, data availability and quality are among the key challenges left to address, where future research could be directed [20]. To enhance cyclone intensity classification, model architecture is one of the most insightful-providing future research directions. ConvLSTM has been confirmed to be effective for capturing spatio-temporal dependencies [45], while developing hybrid deep learning models could further improve performance. For instance, merging ConvLSTM with Transformer-based architectures such as Vision Transformers (ViTs) could enhance the ability to extract long-range features. The dataset would be augmented by providing features to the model that can learn more generalizable patterns. Additionally, future work could incorporate Monte Carlo dropout during inference to estimate predictive uncertainty through multiple stochastic forward passes.

Alternatively, Bayesian deep learning techniques, such as variational inference, can be used to model parameter uncertainty. which offers more reliable forecasts for operational meteorology.

Although SMOTE was applied to reduce class imbalance, there is still minimal representation of cyclone intensity categories, particularly Very Severe Cyclonic Storms (VSCS) or above, contributing to a classification bias and thereby decreasing model sensitivity to these potential high-impact events. Model underrepresentation in severe cyclone categories leads to the misclassification of severe cyclones, which is detrimental to early warning systems. Perhaps the next step can help alleviate this feature underrepresentation through more advanced handling of imbalance such as using adaptive synthetic oversampling, cost sensitive learning, or category specific weighted loss functions. A future consideration could also be to develop either class-conditional synthetic data generation using GANs or variational autoencoders to produce realistic storm examples from the rare categories, which could better support model robustness and recall on the severe cyclone categories. A detailed consideration of spatial, temporal, and other environmental factors would assist in developing a thorough presentation of cyclone behaviour and provide for better accuracy in forecasting. Another problem any studied cyclone will face is the temporal resolution of images taken by satellites. Due to time intervals set when taking photographs, satellite images may fail to capture rapid fluctuations in cyclone intensity due to their limited temporal resolution. Future studies can contrast proposed higher-frequency satellite images, probable over-spatial analyses, or interpolation methods that would help assess intensity variation between images. This usually helps bring out better representation for cyclone evolution in a continuous sense, hence increasing predictive modelling.

To broaden the applicability of the proposed model, future research should explore its adaptability to other ocean basins such as the Atlantic and Pacific. Variations in satellite sensors, image resolutions, and distinct atmospheric dynamics across basins can introduce distributional differences between source and target datasets. Transfer learning and domain adaptation techniques can be employed to fine-tune models trained on North Indian Ocean data for use in other basins, thereby increasing their generalizability and operational value. Another major concern is modelling generalisation across different ocean basins. Cyclone characteristics vary in various regions due to differences in oceanic and atmospheric conditions. Models trained on North Indian Ocean cyclone data may not generalise well to cyclones in the Atlantic or Pacific basins due to regional climatic differences. Domain adaptation and transfer learning techniques can be explored to overcome this [45]. These approaches let you fine-tune the model from one region’s data into another so it’s more equipped for different climatic conditions.

Despite advancements in deep learning, computational complexity remains a challenge. Training ConvLSTM-based models is computationally intensive, and their high inference cost poses challenges for real-time deployment [20]. Such inference currently relies on heavy computation and cannot be operational because swift and efficient prediction is always desired. To address the feasibility of real-time deployment, future work can explore model compression techniques, such as pruning or quantization, and adopt lighter architectures like MobileNet-based ConvLSTM variants. Additionally, real-time forecasting can benefit from deploying the trained model on edge devices or cloud-integrated platforms, which enable low-latency predictions critical for disaster management.

In terms of integration into operational pipelines, future studies must evaluate not just model accuracy but also latency, scalability, and ease of deployment under varying environmental conditions. Solutions like edge deployment, modular architectures, and scalable cloud computing environments can facilitate this integration. Facing these challenges and gazing ahead into future advancements, cyclone classification and intensity estimation models would eventually become more certain and reliable. Integration of advanced deep learning techniques, multi-modal data fusion, domain adaptation, and real-time deployment strategies will add great value to the accuracy and usability of these models. These developments will further contribute to efficient early warning systems, incentives to work towards proactive measures, and a consequent reduction in the impact of cyclones on vulnerable communities. May AI-driven cyclone forecasting lead to great insights into new horizons, greatly transforming the circulation and tracking of storms from the eyes of meteorologists. Bridging current gaps and improving existing models will enable researchers to develop cyclone monitoring systems capable of real-time application.

## Conclusion

This research provides a cutting-edge deep learning model and procedure for detecting, segmenting, estimating intensity, and classifying cyclones using satellite images. Transfer learning models (VGG16, ResNet50, and InceptionV3) were analyzed for a comparative performance whereby VGG16 had the highest accuracy of 99.16%, which was attributed to better feature extraction. Mean Shift achieved a higher Silhouette Score of 0.7772 during cyclone segmentation in comparison to K-Means, thereby demonstrating its suitability of being applied for cyclone isolation. ConvLSTM was used for cyclone intensity estimation and classification, where the clustering-based dataset obtained improved generalization. The model trained on the Mean Shift-clustered dataset produced a RMSE of 8.31 ± 1.10 whereas K-Means had **7.79 ± 1.27**, implying a better RMSE in intensity estimation. While Meanshift based clustering provided better distinction between cyclone intensity levels at 81.1 ± 4.33%, K Mean performed better in intensity estimation. Other challenges such as data imbalance, computational complexity, and model generalization across different ocean basins still derive further advancement in the study. This study proposes future research directions, including hybrid deep learning models combining ConvLSTM and Vision Transformers coupled with Bayesian deep learning for uncertainty estimation and domain adaptation techniques for better generalization across different climatic conditions. Moreover, the applications of lightweight architectures combined with cloud implementations can improve the real-time forecasting of AI-driven cyclone prediction to practically feasible operational levels. With the advent of these primary challenges and the application of ascendant AI-based techniques, cyclone monitoring and forecasting should be prioritized in future meteorological research. Such advancements will contribute in no small way to increased accuracy in early warning systems, hence achieving better disaster preparedness and mitigation, significantly reducing the adverse effects of tropical cyclones on vulnerable communities.
